# Structuring the Future of Cultured Meat: Hybrid Gel-Based Scaffolds for Edibility and Functionality

**DOI:** 10.3390/gels11080610

**Published:** 2025-08-03

**Authors:** Sun Mi Zo, Ankur Sood, So Yeon Won, Soon Mo Choi, Sung Soo Han

**Affiliations:** 1 School of Chemical Engineering, Yeungnam University, Gyeongsan 38541, Republic of Korea; sunmizo@yu.ac.kr (S.M.Z.); ankursood02@gmail.com (A.S.); soyeon1005@ynu.ac.kr (S.Y.W.); 2 Research Institute of Cell Culture, Yeungnam University, Gyeongsan 38541, Republic of Korea

**Keywords:** cultured meat, gel, edible scaffolds, natural polymers, hydrogel

## Abstract

Cultured meat is emerging as a sustainable alternative to conventional animal agriculture, with scaffolds playing a central role in supporting cellular attachment, growth, and tissue maturation. This review focuses on the development of gel-based hybrid biomaterials that meet the dual requirements of biocompatibility and food safety. We explore recent advances in the use of naturally derived gel-forming polymers such as gelatin, chitosan, cellulose, alginate, and plant-based proteins as the structural backbone for edible scaffolds. Particular attention is given to the integration of food-grade functional additives into hydrogel-based scaffolds. These include nanocellulose, dietary fibers, modified starches, polyphenols, and enzymatic crosslinkers such as transglutaminase, which enhance mechanical stability, rheological properties, and cell-guidance capabilities. Rather than focusing on fabrication methods or individual case studies, this review emphasizes the material-centric design strategies for building scalable, printable, and digestible gel scaffolds suitable for cultured meat production. By systemically evaluating the role of each component in structural reinforcement and biological interaction, this work provides a comprehensive frame work for designing next-generation edible scaffold systems. Nonetheless, the field continues to face challenges, including structural optimization, regulatory validation, and scale-up, which are critical for future implementation. Ultimately, hybrid gel-based scaffolds are positioned as a foundational technology for advancing the functionality, manufacturability, and consumer readiness of cultured meat products, distinguishing this work from previous reviews. Unlike previous reviews that have focused primarily on fabrication techniques or tissue engineering applications, this review provides a uniquely food-centric perspective by systematically evaluating the compositional design of hybrid hydrogel-based scaffolds with edibility, scalability, and consumer acceptance in mind. Through a comparative analysis of food-safe additives and naturally derived biopolymers, this review establishes a framework that bridges biomaterials science and food engineering to advance the practical realization of cultured meat products.

## 1. Introduction

The escalating global demand for sustainable and ethical food production has stimulated the development of innovative alternatives to conventional livestock systems. Among these, cultured meat, also known as lab-grown or cell-based meat, has emerged as a promising technological approach with the potential to significantly reduce the environmental footprint, enhance animal welfare, and improve food safety. Produced by cultivating animal cells in vitro under controlled conditions, cultured meat bypasses the need for animal slaughter while offering a promising solution to reduce greenhouse gas emissions, land use, and water consumption associated with traditional meat production [1,2].

**Figure 1:**
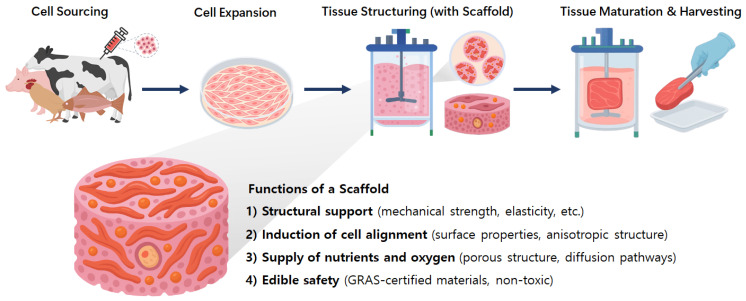
Schematic of cultured meat production highlighting the multifunctional role of scaffolds in tissue structuring, nutrient delivery, and ensuring edibility.

Despite its conceptual appeal, the successful commercialization of cultured meat remains contingent upon overcoming several technical and economic challenges. One of the most formidable among these is the replication of the structural, mechanical, and sensory attributes of conventional meat. While ground meat products can be relatively easily mimicked through unstructured cell masses, structured meat analogs such as steaks, chicken breasts, or pork loins require the formation of spatially organized, three-dimensional constructs composed of aligned muscle and fat tissues. Achieving such complexity necessitates the incorporation of scaffolding systems that can support cell proliferation, guide tissue formation, and ultimately contribute to the texture and integrity of the final product [3,4]. Schematic representation of cultured meat production is represented in Figure 1.

In cultured meat production, scaffolds serve not merely as passive supports but as bioactive architectures that actively regulate cellular behavior [3]. They provide a framework for cell adhesion, proliferation, differentiation, and alignment, which are crucial for the development of organized muscle fibers. Moreover, scaffolds enable the diffusion of oxygen and nutrients into cell-dense regions, support the removal of metabolic waste, and contribute to the mechanical strength and texture of the mature tissue. Critically, scaffolds intended for food applications must not only satisfy the functional requirements of tissue engineering but must also be edible, non-toxic, and compatible with food regulatory standards [5].

To address these multifaceted demands, growing attention has been directed toward hybrid bio-based composite scaffolds, composed primarily of naturally derived polymers integrated with structural reinforcements or functional additives. In this context, hybrid hydrogel-based scaffolds, referring to gel-forming biopolymer matrices such as gelatin, alginate, or chitosan, combined with structural or functional agents that enhance their mechanical additives, are compounds recognized as safe for consumption that contribute to scaffold reinforcement, cell compatibility, and food processing stability. Biocompatible, biodegradable, and edible materials from various natural sources, such as animal-derived (e.g., collagen, gelatin, fibrin, albumin, and casein), plant protein (e.g., soy, wheat, pea, rice, potato, and corn), algae-derived (e.g., alginate, carrageenan, and agar), plant-based (e.g., cellulose, starch, pectin, and lignin), and crustacean shell-derived (e.g., chitosan, chitin, glucosamine, and calcium carbonate) materials have attracted increasing interest, owing to their features that are required for designing cultured meat (Table 1). However, their inherent limitations, such as poor mechanical strength or limited processability due to factors like thermal instability, poor printability, and scalability issues, have prompted researchers to explore hybridization strategies, in which complementary materials are combined to enhance the scaffold’s physicochemical performance [3,4,6,7].

To function effectively as scaffolds for cultured meat, these composite systems must satisfy a set of stringent design criteria, which are elaborated below: First, biocompatibility is essential to support cell–material interactions and downstream tissue development. Scaffolds must present a surface environment that supports cell adhesion, viability, and differentiation. These biological responses are governed by surface properties such as roughness, hydrophilicity, and surface charge. Myogenic cells, in particular, are highly sensitive to their microenvironment, necessitating finely tuned biochemical and biophysical cues to promote the formation of aligned myotubes and functional muscle tissue [8,9,10].

Second, edibility and food safety are non-negotiable. Unlike biomedical scaffolds, those used in cultured meat must be safe for consumption, digestible, and metabolically inert or beneficial. Common synthetic polymers such as polycaprolactone (PCL) or polylactic acid (PLA), known for their biodegradable and favorable mechanical properties in tissue engineering, are generally unsuitable for edible scaffolds unless proven safe at dietary exposure levels. Instead, materials recognized as safe under GRAS (Generally Recognized As Safe) regulations, primarily applicable within the United States, are prioritized. Equivalent safety assessments are governed by other regulatory bodies such as EFSA in Europe and FSSAI in India. Moreover, the ideal scaffold may even enhance the nutritional profile or organoleptic properties (e.g., texture and flavor) of the final meat product [11,12].

Third, mechanical integrity is essential. The scaffold must maintain its structural integrity throughout the tissue maturation process and withstand mechanical stresses encountered during downstream processing, including slicing and cooking. The elastic modulus of the scaffold should ideally approximate that of native muscle tissue (typically 10–100 kPa). To improve stiffness and elasticity, mechanical reinforcement can be achieved through the incorporation of nanocellulose and zein, respectively, while protein-based crosslinkers may enhance toughness and structural cohesion [13,14].

Fourth, porosity and microarchitecture play critical roles in nutrient and oxygen diffusion, particularly in thick or multilayered tissue constructs, where dense cellular arrangements and limited interstitial space hinder the effective transport of oxygen and nutrients. An optimal scaffold should exhibit interconnected pores ranging from 50 to 200 μm, facilitating efficient mass transport and promoting cell infiltration [15]. Furthermore, anisotropic structures, such as aligned fibers or channels, are advantageous for guiding muscle cell orientation and mimicking the highly ordered architecture of skeletal muscle. Fabrication techniques such as electrospinning, freeze-drying, and 3D bioprinting offer distinct advantages in controlling scaffold morphology, for example, electrospinning is well-suited for producing aligned fibrous structures, while 3D bioprinting enables the creation of complex, spatially defined geometries [17,18].

Finally, scalability and manufacturability must be considered for industrial applications. Scaffold materials must be cost-effective, abundant, and compatible with large-scale production systems, including bioreactors. The fabrication process should be based on mild, non-toxic, and water-based methods, ideally avoiding organic solvents and synthetic crosslinkers to maintain a clean-label profile acceptable to consumers and regulatory bodies [5,16].

In summary, gel-based scaffold development for cultured meat lies at the intersection of biomaterials science, tissue engineering, and food technology. The dual need for biological functionality and food safety presents a unique set of challenges that are being addressed through the design of hybrid gel-based composite materials. This review aims to provide a comprehensive overview of recent advances in this field, covering materials, fabrication strategies, and application-specific considerations that are shaping the future of scalable and edible cultured meat production.

## 2. Hybrid Bio-Hydrogel-Based Composite Materials

The selection of scaffold materials that are both biocompatible and edible is a central challenge in the design of cultured meat systems. To address this, recent research has increasingly focused on hybrid bio-based composites composed of naturally derived polymers and food-grade components [7]. These hydrogel-based composite materials offer a balanced integration of mechanical stability, cellular functionality, and food safety. However, such integration is often difficult to achieve using single-component systems due to inherent trade-offs between properties such as stiffness and degradability [19].

This section begins by examining the roles of structurally functional natural polymers as foundational scaffold matrices. It then explores the synergistic combination of bio-derived and food-compatible additives that can enhance both performance and safety. Finally, we discuss strategies to tailor the physicochemical properties of these materials to optimize their suitability for edible scaffold applications.

### 2.1. Structurally Functional Natural Polymers

Scaffold systems for cultured meat must go beyond merely providing a physical framework for cell adhesion [20]. They must actively contribute to tissue organization, cellular alignment, and ultimately, the development of consumable muscle-like constructs [21]. To fulfill these multifaceted roles, gel-based scaffold materials must exhibit a unique combination of biological functionality, mechanical robustness, edibility, and food safety. In this context, naturally derived polymers represent a promising class of materials that intrinsically satisfy many of these criteria while remaining amenable to further physicochemical tuning.

(1) Gelatin and Collagen

**Figure 2:**
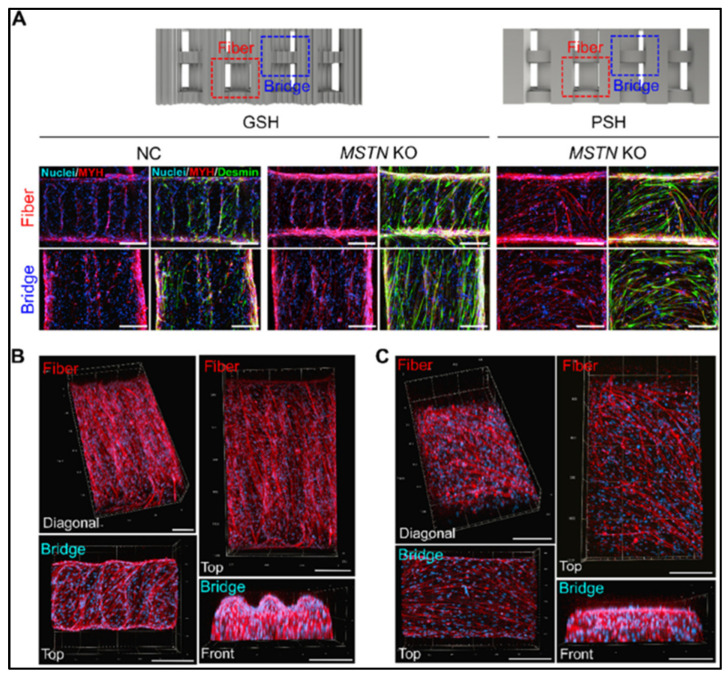
(**A**) Immunofluorescence images of NC and MSTN KO cells grown on GSH and PSH (red = myosin heavy chain MYH; blue = nuclei; green = desmin), 3D construction of MSTN KO myotubes in (**B**) GSH, and (**C**) (PSH) (scale bar =200 µm). Reprinted with permission from [25] (Copyright 2025, BMC).

Gelatin, a partially hydrolyzed form of collagen, has long been recognized for its excellent biocompatibility, food-grade safety, and thermoreversible gelatin behavior [22]. It contains arginine–glycine–aspartic acid (RGD) sequences, which serve as critical ligands for integrin-mediated cell adhesion, promoting the proliferation and myogenic differentiation of cells such as C2C12 myoblasts and preadipocytes. In this regard, Hong et. al. reported augmented growth and proliferation of adipose tissue-derived stem cells (ADSCs) by coating soy protein–agarose scaffolds using gelatin. The presence of RGD (Arg-Gly-Asp) peptide sequences in gelatin, a naturally occurring cell adhesion motif found in ECM proteins, has been shown to significantly enhance cell adhesion, proliferation, and differentiation via integrin-mediated pathways [23,24]. These bioactive motifs not only promote initial cell attachment to the scaffold but also activate intracellular signaling cascades that guide tissue development, highlighting their potential as a functionalization strategy in edible scaffold systems. Upon heating above physiological temperature (~37 °C), gelatin undergoes sol–gel transitions, rendering it suitable not only for tissue formation but also for improving the textural and sensory properties of the final edible product. In a study, reported by Eom et al., multichannel-grooved steak-like hydrogel-based scaffolds were fabricated using GelMA hydrogel bioinks. This was achieved through a 3D printing approach incorporating MSTN knock-out cells [25]. Here, the myogenic differentiation and alignment were demonstrated in grooved-shaped hydrogels (GSH) and plain-shaped hydrogels (PSH). The immunostaining of negative controls (NC) and MSTN knock-out (MSTN KO) cells grown on GSH and PSH was evaluated for fiber and bridged regions with 3D prediction of growth and alignment of the myotubes (Figure 2). Further, in a separate study, Wang et al. demonstrated the utilization of 3D printing in fabricating gelatin/alginate/poly-l-Lysin gel-based scaffolds for porcine muscle stem cell expansion while targeting cultured meat production [26].

**Figure 3:**
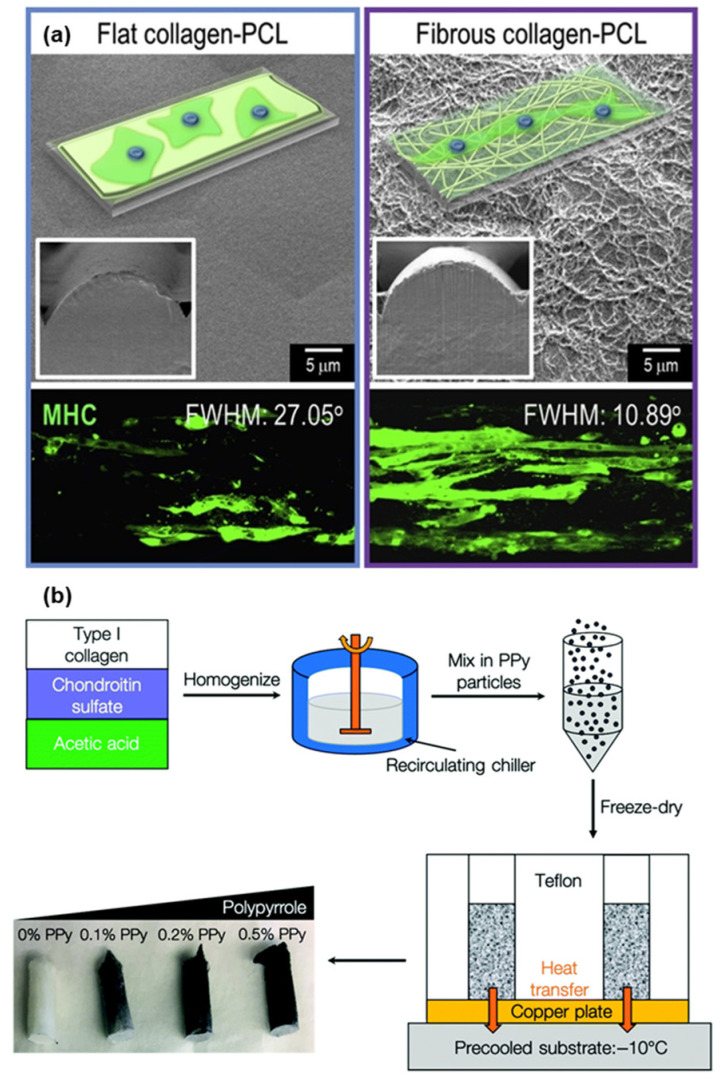
(**a**) Schematic representation of flat collagen PCL and fibrous collagen PCL scaffolds along with their immunofluorescent images after 14 days of culture (MHC = green). Reprinted with permission from [27] (Copyright 2019, Elsevier). (**b**) Schematic representation of the collagen-glycosaminoglycan–polypyrrole (CG-PPy) scaffolds via a directional lyophilization approach. Reprinted with permission from [29] (Copyright 2021, RSC).

Collagen, particularly types I and II derived from porcine or piscine sources, is structurally analogous to native extracellular matrix (ECM) components in skeletal muscle. Its fibrillar architecture and biological recognition motifs support muscle cell alignment and fusion into multinucleated myotubes. In this regard, Chae et al. reported the formation of skeletal myotubes on fibrillated collagen nanofiber-coated polycaprolactone (PCL) struts fabricated via 3D printing. The PCL scaffold featured a grooved surface, and the fibrillated collagen coating enhanced C2C12 myoblast alignment and elongation compared to non-fibrillated controls, indicating that the nanoscale topography provided effective cues for myotube formation [27]. Here, three different densities of collagen/fibrin were selected to coat the surface of PCL and were investigated for the activity, growth, and proliferation of myoblasts (Figure 3a). Additionally, Liu et al. reported the utilization of type 1 collagen in promoting the migration and differentiation of C2C12 myoblasts [28]. Numerous studies have demonstrated the efficacy of collagen-based hydrogels and sponges in guiding muscle tissue morphogenesis, while Basutro et al. reported the effect of electrically conducted 3D collagen scaffolds in aligning skeletal muscles [29]. In this study, a mixture of type 1 collagen, chondroitin sulfate, and acetic acid was homogenized and mixed with polypyrrole (PPy), and aligned scaffolds were fabricated via directional lyophilization (Figure 3b). Yun et al. demonstrated the efficacy of collagen gel-based matrices functionalized using fibroblast growth factor 2 as a potent candidate in skeletal muscle growth and alignment [30]. However, both gelatin and collagen suffer from limited mechanical strength, high water uptake, and shape instability under prolonged culture conditions [31,32]. As such, they are often crosslinked chemically or physically, or blended with structurally robust polymers to enhance their functional performance [33,34].

(2) Chitosan

Chitosan is a cationic polysaccharide obtained through the deacetylation of chitin, typically sourced from crustacean shells or insect cuticles. Reported degrees of deacetylation vary depending on the source and processing method, ranging from 67% in insect-derived chitosan to 92% in shrimp-derived chitosan. This parameter significantly influences functional properties such as solubility, charge density, and biocompatibility [35]. It is biodegradable, biocompatible, antimicrobial, and film-forming, making it particularly advantageous for scaffold applications in long-term cultures. In aqueous environments below pH 6.5, chitosan carries a positive surface charge, enabling electrostatic interactions with negatively charged cell membranes, which in turn enhances cell adhesion [36].

**Figure 4:**
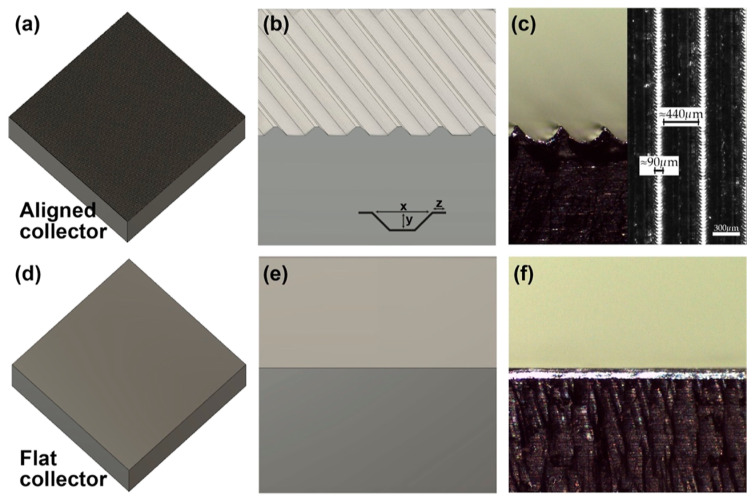
Schematic representation of the MP collector plates. (**a**,**b**) Top view of the MP collector plate; (**c**) sideview of the aligned collector with channel width as x ≈ 440 µm, y ≈ 160 µm, and z ≈ 90 µm; (**d**,**e**) top view of the flat (control) collector plate; and (**f**) sideview of the control plate. Reprinted with permission from [37] (Copyright 2023, ACS).

Electrospun freeze-dried chitosan scaffolds have been shown to support 3D cell growth and fiber alignment, especially when seeded with myoblasts or satellite cells. A combination of electrospinning and 3D printing of gelatin, chitosan, and PVA scaffolds has been studied by Taborda et al. for maintaining muscle alignment and growth [37]. In one approach, a micropatterned (MP) collector was used to fabricate nanofibrous scaffolds with varied compositions in order to investigate the resulting structural and functional properties of the materials. In the MP collector, the topological features were controlled with a computer numerical control (CNC) milling machine (Figure 4). Further, Smoak et al. highlighted the potential of an electrospun decellularized skeletal muscle extracellular matrix in controlling the rat-based myoblast proliferation and myotube formation [38]. Additionally, the utility of electrospun chitosan microfibers to support the attachment and viability of rat muscle-derived stem cells was highlighted by Kang et al. [39]. Moreover, Ma et al. fabricated chitosan-grafted aniline electrospun nanofibers and demonstrated their ability for cell adhesion and proliferation of C2C12 myoblasts and dog chondrocyte cells [40]. Furthermore, its inherent broad-spectrum antimicrobial properties, effective against both bacteria and fungi, help reduce contamination risks during extended cultivation [41]. Nonetheless, the use of chitosan in food applications requires careful consideration due to its potential allergenicity and its derivation from shellfish, which may raise regulatory and consumer concerns in certain markets [42].

(3) Cellulose and its derivatives

**Figure 5:**
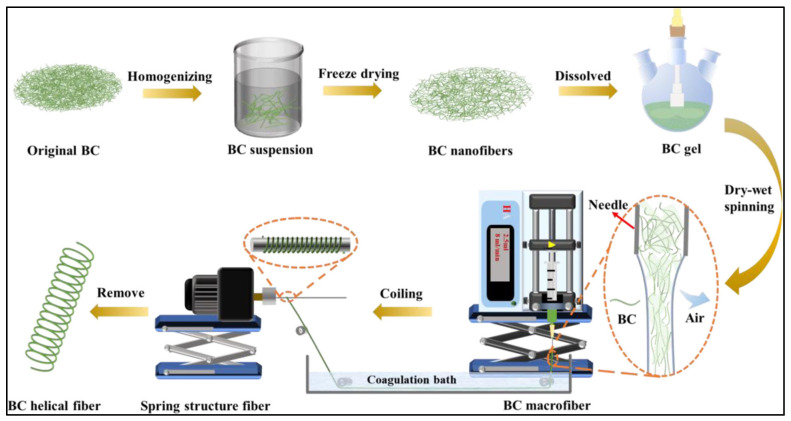
Schematic representation of the fabrication of BC-based helical fibers. Reprinted with permission from [44] (Copyright 2021, ACS).

Cellulose, the most abundant biopolymer on Earth, offers exceptional mechanical integrity, environmental sustainability, and scalability [43]. Its nanostructured forms, such as cellulose nanofibers (CNFs) and cellulose nanocrystals (CNCs), are particularly valuable in cultured meat scaffolds due to their ability to form high-strength, anisotropic matrices that can direct cell alignment and mimic the fibrous architecture of skeletal muscle. In this regard, Liang et al. demonstrated that cellulose fibers fabricated through precise molecular packing exhibited superior transverse tensile strength compared to natural fibers, suggesting the potential of molecular-level structural control strategies for the development of high-strength biofibers [44]. In this study, a dry–wet spinning method was used to fabricate bacterial cellulose (BC)-based macrofibers with very high strength, with the ability to be developed as a superstretchable helical fiber structure. (Figure 5). Further, the utilization of cellulose nanofibers for cell alignment has been reported by Skogberg et al., where it was demonstrated that the contact dispersion technique of droplet-evaporated cellulose nanofibers could regulate direct cell alignment of cells on the scaffolds [45]. In another study, Murugarren et al. provided a straightforward approach for cell alignment on aligned BNCs using a chain of biochemical reactions [46]. Cellulose has been explored to imitate the fibrous architecture of skeletal muscles, as reported by Santos et al., where annatto-loaded bioactive cellulose nanofibers were used to imitate and support skeletal muscle cells’ growth and proliferation [47]. Mastodimos et al. demonstrated the potential of BMC-based fibers to support aligned myoblast growth [48].

However, native cellulose and its nanofiber derivatives exhibit low cell adhesion due to their intrinsic hydrophilicity and lack of adhesive ligands, despite offering structural stability and biocompatibility. Thus, while cellulose serves as a robust physical framework, its limited bioactivity may hinder direct cell–material interactions [44,49,50,51]. To overcome these limitations, surface modifications such as TEMPO-mediated oxidation [52], amination [53], or silanization [54] have been employed to enhance cell compatibility. Specifically, TEMPT-mediated oxidation introduces carboxyl groups that improve hydrophilicity and cytocompatibility, amination provides amino functionalities facilitating hydrogen bonding and potential biofunctionalization, while silanization with thiol-functional siloxanes enhances cell adhesion by tuning surface chemistry and wettability. Moreover, cellulose derivatives, including cellulose acetate (CA) and carboxymethyl cellulose (CMC), are approved for food use and are frequently incorporated to balance edibility with structural reinforcement [55]. These derivatives also facilitate blending with gelatin [56], chitosan [57], or alginate to create multifunctional gel-based scaffold matrices.

(4) Plant-derived proteins

**Figure 6:**
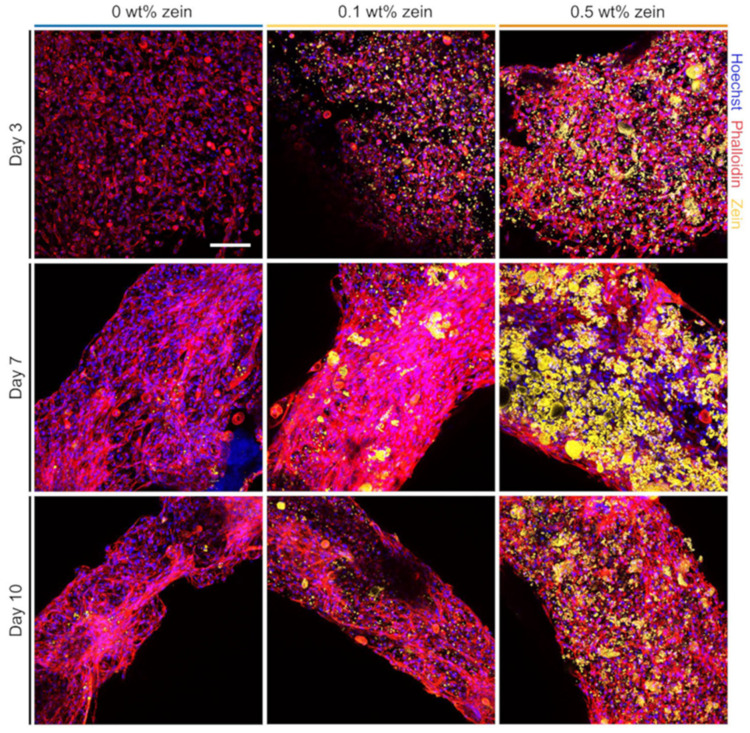
Fluorescent images demonstrating bioartificial muscle (BAM) morphology with C2C12 cells on days 3, 7, and 10 (Phalloidin = red; zein = yellow; Hoechst = blue, scale bar = 100 µm). Reprinted with permission from [58] (Copyright 2023, MDPI).

Plant-based proteins are gaining increasing attention in cultured meat scaffold design due to their renewability, nutritional value, and wide acceptance as food ingredients. Among them, soy protein isolate (SPI), pea protein, and zein stand out as particularly promising. Zein, a prolamin extracted from corn, is characterized by its amphiphilic block structure, which enables the formation of stable fibers and films via electrospinning. As a relevant case study, Melzener et al. fabricated aligned scaffolds by weaving alginate fibers coated with zein, followed by a stretching process [58]. When C2C12 myoblasts were cultured on these scaffolds, enhanced cell alignment and myotube formation were observed (Figure 6). These results suggest that the physical architecture of the fibers can effectively guide cellular behavior. The scaffold demonstrated both edibility and functional applicability, highlighting its potential for use in cultured meat production. In a related effort, Kawecki et al. developed an edible electrospun nanofiber scaffold composed of zein and gelatin and cultured C2C12 myoblasts on the structure. The results showed high cell viability and the formation of aligned myotubes, with the woven fiber structure further promoting tissue organization. Additionally, cooking tests conducted on the cell–fiber composite confirmed its ability to partially replicate the appearance and texture of conventional meat [59]. Its hydrophobicity and resistance to enzymatic degradation further contribute to its mechanical resilience in dynamic culture environments.

Soy protein isolate, on the other hand, exhibits excellent gelation and thermal crosslinking properties, allowing for the fabrication of stable, food-grade scaffolds through extrusion or 3D bioprinting. SPI-based hydrogels have been investigated as edible bioinks, supporting cell viability while maintaining shape fidelity during long-term culture. Dey et al. developed an edible bioink based on SPI and used it to fabricate 3D-printed scaffolds with a lattice-like and multilayered structure. These constructs exhibited high mechanical stability, shape fidelity, and potential applicability in cultured meat production. The printed gel constructs exhibited high shape fidelity and mechanical stability, and their suitability as food materials and processability were evaluated, demonstrating their potential for applications in cultured meat production [60]. Similarly, Kim’s research team evaluated the 3D printability and potential application of a composite gel composed of pectin blended with soy protein isolate (SPI) and pea protein as a scaffold for cultured meat. The resulting gel-based scaffolds exhibited rheological properties such as shear-thinning behavior and structural recovery, and the addition of proteins was found to enhance printability and improve textural properties [61]. However, plant proteins are often sensitive to processing conditions such as humidity and temperature and may lack sufficient mechanical strength or pore-forming capacity when used alone. As such, they are frequently combined with cellulose or alginate to create composite scaffolds with improved performance.

(5) Other food-compatible polysaccharides

Additional polysaccharides such as alginate and agarose, derived from marine algae, have been widely utilized in food and biomedical applications. Alginate forms ionically crosslinked hydrogels in the presence of divalent cations like Ca^2+^, offering mild gelation conditions, excellent biocompatibility, and ease of handling. Seo and colleagues proposed the potential application of alginate-based scaffolds with precisely controlled crosslinked structures for cultured meat production, demonstrating improved cell adhesion and the formation of aligned muscle cell networks [62]. Similarly, Li et al. developed an edible three-dimensional scaffold composed of chitosan, alginate, and collagen/gelatin, which supported the culture of porcine skeletal muscle satellite cells. The scaffold exhibited excellent biocompatibility and promoted cell adhesion. After 14 days of culture, enhanced myogenic differentiation was confirmed by increased expression of MyoD, Myogenin (MyoG), and Myosin Heavy Chain (MyHC), as determined by qPCR and Western blotting [63]. However, its lack of intrinsic cell-adhesive sequences necessitates blending with bioactive proteins (for example, gelatin or collagen) for effective tissue formation.

Agarose, a thermoreversible polysaccharide, allows for the creation of structurally stable scaffolds upon cooling, but its low mechanical strength and poor cell-adhesive properties limit its use as a primary matrix material. Therefore, it is typically employed as a supporting component within hybrid composite systems.

Collectively, structurally functional natural polymers serve as biologically active, edible platforms that guide tissue formation while ensuring safety for human consumption. Each polymer class offers distinct advantages in terms of biological interaction, mechanical profile, processing compatibility, and regulatory status. Their rational selection, modification, and combination are essential to fabricate scaffolds that not only support muscle and fat tissue development but also contribute to the textural and nutritional quality of the final cultured meat product. In the subsequent section, we examine how these base polymers can be further enhanced through synergistic incorporation of bio-derived and food-grade additives, enabling the design of hybrid composite scaffolds that meet the complex demands of cultured meat engineering.

### 2.2. Synergy Through Bio-Derived and Food-Grade Additives

Cultured meat scaffolds are uniquely required to meet both biocompatibility and edibility criteria, which poses significant material design challenges. While naturally derived polymers provide a biologically favorable foundation, they often lack the necessary mechanical robustness, cellular guidance capability, and processability when used alone. To overcome these limitations, recent research has increasingly focused on integrating food-grade additives and bio-derived functional materials with natural polymer matrices to enhance scaffold performance through synergistic interactions.

(1) Nanocellulose and Cellulose-derived reinforcements

Nanocellulose, encompassing both cellulose nanofibers (CNFs) and cellulose nanocrystals (CNCs), has emerged as a structurally potent additive due to its high tensile strength (>2 Gpa), low density (~1.6 g/cm^3^), and exceptionally large surface area (>100 m^2^/g). These properties enable nanocellulose to serve as an effective reinforcing agent when embedded within soft biopolymeric matrices. When incorporated into natural polymer scaffolds such as gelatin [64], zein [65], or alginate [66], nanocellulose has been shown to improve multiple performance parameters:

**Mechanical enhancement:** Incorporation of 0.1–1.0 wt% cellulose nanofibers (CNFs) into gelatin matrices has been reported to enhance tensile strength by more than 1.3-fold and increase the elastic modulus by approximately 40% [64]. This enhancement is attributed to the ability of cellulose fibers to dissipate stress and reduce crack propagation within the polymeric network.

**Cellular alignment support:** Aligned CNF structures or nanofiber-reinforced scaffolds fabricated via directional assembly have demonstrated the ability to promote nuclear alignment and myotube formation of muscle cells, including C2C12 myoblasts [67]. Electrospun zein/nanocellulose composite scaffolds have also been reported to facilitate cell alignment. Wan et al. developed cellulose–zein composite nanofiber membranes through an eco-friendly self-assembly process and demonstrated that the zein-modified nanofibers significantly enhanced cell adhesion and proliferation, attributed to the increased surface roughness and excellent biocompatibility of zein [68].

**Regulatory and clean-label compatibility:** Several cellulose derivatives including carboxymethyl cellulose (CMC), microcrystalline cellulose (MCC), and hydroxypropyl methylcellulose (HPMA) are recognized as food additives under FAO/WHO JECFA (Joint FAO/WHO Expert Committee on Food Additives) and have a GRAS (Genaerally Recognized As Safe) status by the FDA [69]. Their inclusion in scaffold formulations not only enhances mechanical stability and printability but also aligns with clean-label requirements for food applications. Additionally, these materials may serve a dual purpose by contributing dietary fiber functionality to the final product.

Together, these cellulose-based additives exemplify how the integration of structurally competent, food-safe reinforcements can substantially elevate the performance of edible scaffolds, bridging the gap between biomimetic functionality and regulatory compliance in cultured meat systems.

(2) Natural crosslinkers and functional binders

In the development of edible gel-based scaffolds for cultured meat, natural crosslinkers and functional binders have gained considerable attention as effective strategies for overcoming the intrinsic limitations of unmodified biopolymer matrices [70]. These additives enhance scaffold performance by promoting covalent or non-covalent interactions between polymer chains, thereby improving mechanical strength, thermal stability, and structural integrity, all while remaining compliant with food safety regulations [71].

**Transglutaminase (TGase)** is an enzyme-based crosslinker widely utilized in the food industry. It catalyzes the formation of ε-(γ-glutamyl) lysine isopeptide bonds between glutamine and lysine residues in proteins, facilitating the crosslinking of protein chains [72]. When applied to gelatin, soy protein, or casein-based scaffold systems, TGase forms a denser polymeric network, leading to marked improvements in tensile strength, elasticity, and thermal resistance [73]. For example, TGase-treated gelatin gels have been reported to exhibit improved mechanical stability and thermal degradation resistance, making them suitable for long-term culture conditions [74]. Chien and colleagues demonstrated that gels made from soy protein treated with microbial transglutaminase (mTGase) showed an enhanced compressive modulus ranging from 50 to 100 Pa, while also reducing the degradation rate, thereby improving mechanical and structural stability [75]. Sengor’s group utilized microbial transglutaminase (mTGase) to crosslink sodium caseinate (a casein derivative) and starch-based composite hydrogel scaffolds, achieving a high tensile strength of 690 kPa in the dry state and effectively promoting cell adhesion and proliferation [76]. Furthermore, TGase is GRAS-certified by the FDA and approved by multiple international regulatory bodies, making it one of the most promising crosslinking agents for edible scaffolds in cultured meat applications [77].

**Polyphenolic compounds,** such as tannic acid, catechins, and gallic acid derivatives, are highly reactive with amine and hydroxyl groups and can serve as natural crosslinkers or surface modifiers. These molecules reinforce interpolymeric bonding through hydrogen bonding and oxidative covalent interactions, thereby improving both mechanical robustness and water resistance [78]. For instance, tannic acid has been shown to enhance the mechanical stability of chitosan and gelatin-based scaffolds while simultaneously improving cell adhesion by modulating surface hydrophilicity. In addition, their antioxidant properties may extend the shelf life and oxidative stability of the final cultured meat product, offering functional benefits beyond structural support. This is supported by the findings of Taheri et al., who demonstrated that the incorporation of tannic acid into chitosan/gelatin gel films significantly improved mechanical properties, reduced water vapor permeability, and enhanced in vivo wound healing efficacy, suggesting its translatability to tissue-mimetic edible scaffolds for cultured meat applications [79].

**Plant-derived viscous biopolymers,** including mucilage, gum arabic, and guar gum, are multifunctional ingredients that contribute to viscosity modulation, elasticity adjustment, and moisture retention [71]. When blended with protein-based gel matrices, these polysaccharides can fine-tune gelation behavior, improve printability, and enhance shape retention during scaffold fabrication [80]. For example, hydrogel systems combining gum arabic and gelatin have demonstrated excellent thermal stability and extrudability, making them suitable for use in 3D printing-based scaffold construction. Importantly, these biogums are widely accepted as food-safe, and their cultural familiarity and regulatory clearance in diverse markets enhance their potential for commercial adoption [81,82].

In summary, natural crosslinkers and binders play a pivotal role in bridging the gap between functional performance and regulatory compliance. Their integration into biopolymer-based systems enables the fabrication of robust, bioactive, and food-compatible scaffold platforms, advancing the technological readiness of cultured meat production.

(3) Dietary fibers, modified starches, and rheological modifiers

In the context of cultured meat production, scaffolds must function not only as physical supports for cells but also satisfy key engineering requirements, including precise structural design, processing adaptability, and long-term dimensional stability. In particular, 3D shape retention, pore architecture maintenance, and printability are essential not only for uniform tissue growth but also for ensuring scalability and manufacturability in commercial applications. To address these multifaceted needs, recent research has increasingly focused on the use of plant-derived functional polysaccharides, notably dietary fibers, modified starches, and rheological modifiers, as promising multifunctional additives capable of tuning both the physical and biological properties of edible scaffolds [83,84,85,86,87].

Dietary fibers are generally categorized into insoluble fibers (ex., cellulose, hemicellulose, and lignin) and soluble fibers (ex., inulin, β-glucan, and pectin), each contributing to the scaffold functionality in distinct ways. In the case of insoluble fibers, they reinforce scaffold matrices by acting as filler materials and fiber-based networks, enhancing the mechanical strength and elasticity of biopolymer-based scaffolds such as those composed of gelatin, alginate, or zein [68]. In particular, lignin and hemicellulose increase interchain bonding density within the matrix, which improves resistance to shrinkage and swelling. This enhanced dimensional stability supports shape retention during long-term culture and improves printing fidelity in biomanufacturing processes such as 3D bioprinting [88]. Soluble fibers, on the other hand, increase viscosity and induce gelation in moist environments, thereby improving pore stability and diffusional transport of nutrients and oxygen within the scaffold. β-glucan, in particular, is known for its immunomodulatory and antioxidant activities, which help establish a physiologically favorable microenvironment for cell survival [89]. Additionally, these soluble polysaccharides enhance water-holding capacity, which is crucial for maintaining the hydrogel-like integrity of edible scaffolds. Moreover, most dietary fibers are classified as GRAS by the U.S. FDA and have a long history of use in food products, providing excellent regulatory compliance and consumer acceptability. Their high compatibility with plant proteins, cellulose, and starch also makes them ideal for composite scaffold formulations [12,85].

Modified starches, obtained by physical, chemical, or enzymatic modification of native starch, offer further functionality for the scaffold system, including viscosity tuning, gel strength enhancement, and improved thermal stability. Pregelatinized starch, for example, can gel in the presence of water alone and forms stable gels even at room temperature, contributing to viscoelastic control and prevention of collapse in printed structures [90]. Acetylated starch exhibits improved heat and storage stability, enabling shape maintenance during long-term culture and post-processing steps such as washing, harvesting, and thermal cooking [91]. Oxidized starch, which contains reactive aldehyde groups, may be used to promote chemical crosslinking within the scaffold matrix or serve as a carrier platform for bioactive signaling molecules. Moreover, it facilitates fine-scale pore generation, which is beneficial for tissue permeability [92]. These starch derivatives are also well-suited for high-throughput fabrication processes, including extrusion, molding, and spray drying, offering practical advantages in terms of manufacturing scalability and cost reduction.

Rheological modifiers are crucial in scaffold fabrication using bioprinting platforms, as they directly influence material flow behavior, gelation kinetics, and shape fidelity. Carboxymethyl cellulose (CMC), a water-soluble cellulose derivative, excels in viscosity control, gel stabilization, and moisture retention [93]. Its strong shear-thinning behavior makes it highly suitable for high-resolution printing, while also supporting volumetric stability of the scaffold during culture. Hydropropyl methylcellulose (HPMC), widely used in food and pharmaceutical formulations, exhibits thermal gelation properties, enabling shape retention under heating conditions such as cooking. When combined with proteins like zein or gelatin, HPMC improves internal structural integrity and post-printing curing stability [94]. These cellulose-based rheological agents are predominantly GRAS-certified and offer essential structural and material consistency in the development of 3D printable scaffold inks [95].

In summary, dietary fibers, modified starches, and rheological modifiers represent a class of multifunctional plant-based additives capable of meeting the complex demands of cultured meat scaffolds. They effectively address requirements related to mechanical robustness, microstructural fidelity, manufacturing performance, and food safety. Particularly in the context of commercial-scale, ready-to-cook cultured meat products, these materials offer strategic value by complementing the limitations of conventional biopolymers and serving as the core of functional hybrid scaffold design.

## 3. Summary and Outlook

Cultured meat represents a transformative approach to sustainable food production, offering solutions to environmental, ethical, and food security challenges. A critical technological component enabling its success lies in the development of edible gel-based scaffolds capable of supporting the growth, organization, and maturation of animal cells into structured, meat-like tissues. This review has comprehensively explored recent strategies in the design of such scaffolds using hybrid gel-based composites, highlighting the interplay between material composition, functional performance, and regulatory viability.

Effective scaffold systems must concurrently meet five essential criteria: biocompatibility, edibility, mechanical stability, porosity, and manufacturability. Natural polymers such as gelatin, collagen, chitosan, cellulose, zein, and plant-derived proteins form the basis of gel-based scaffolds due to their inherent bioactivity and safety. These are often complemented by functional, food-grade additives including nanocellulose, dietary fibers, modified starches, transglutaminase, polyphenols, and rheological modifiers (ex., CMC and HPMC) to enhance structural integrity, printability, and cell compatibility. Hybridization strategies allow for synergistic tailoring of scaffold properties, enabling the integration of mechanical support, nutritional compatibility, and consumer safety in a single platform. Despite these advancements, several technical and industrial limitations persist. Trade-offs between mechanical robustness, digestibility, and textural fidelity remain difficult to optimize. The long-term stability of scaffolds under dynamic culture conditions, interactions between multiple bio-based components, and the reproducibility of complex architectures require further systematic investigation. Moreover, the lack of standardized performance data and predictive design tools limits the ability to rationally engineer scaffold formulations for scale-up.

To bridge these gaps, future research must address the following:

- Quantitative structure–function mapping between scaffold composition, microarchitecture, and cellular behavior (ex., proliferation, alignment, and differentiation);
- Mechanistic insights into material–cell interactions to inform bioinspired scaffold design;
- Advanced hybridization strategies that optimize the trade-offs between printability, structural fidelity, and sensory performance;
- Integration with bioprinting process parameters to enable precision fabrication of edible, tissue-like constructs.

In parallel, the scale-up, regulatory compliance, and commercialization of edible scaffolds present distinct challenges;

- Scale-up: Material formulations optimized at the lab scale may not be directly transferable to industrial bioreactors. Scaffold systems must be engineered to withstand long-term immersion in culture media while maintaining geometry, porosity, and bioactivity. Compatibility with high-throughput fabrication processes, including extrusion, molding, and 3D printing, will be essential for cost-effective production.
- Regulatory consideration: All scaffold components must comply with food safety standards, such as FDA GRAS or EFSA Novel Food requirements. Ingredients like chitosan, zein, or functionalized polysaccharides may require thorough evaluation of toxicity, allergenicity, metabolic impact, and residual presence in the final product. Therefore, the establishment of a standardized framework for evaluating scaffold safety, functionality, and nutritional impact is urgently needed.
- Commercial prospects: While scaffolds may contribute a relatively small portion of production costs, they exert a disproportionately large influence on product texture, structure, cookability, and consumer acceptability. The next generation of scaffolds must be designed not only to support cell growth but also to align with culinary expectations, enabling the fabrication of premium, structured meat analogs with palatable texture and sensory appeal.

Hybrid gel-based composite scaffolds represent a foundational technology in cultured meat production, bridging the gap between tissue engineering, food science, and sustainable manufacturing. The strategies and material platforms outlined in this review provide a roadmap toward the development of scalable, safe, and consumer-ready scaffold systems. As the cultured meat industry moves closer to commercialization, such scaffold innovations will be key to realizing its full potential as a next-generation protein source in the global food system.

## 4. Conclusions

Cultured meat is increasingly recognized as a pivotal technology in the transition toward sustainable food systems. Its commercial viability, however, hinges critically on the development of edible scaffolds that satisfy both biological functionality and food safety requirements. This review has focused on recent advancements in the design of such scaffolds using hybrid gel-based composites, highlighting how the combination of natural polymers and food-grade functional additives can address the multifaceted demands of structural stability, biocompatibility, and processability.

Naturally derived materials such as gelatin, chitosan, cellulose, and plant-based proteins provide a biocompatible and edible foundation, while the incorporation of nanocellulose, dietary fibers, modified starches, enzymatic crosslinkers, and rheological modifiers enables the development of multifunctional scaffold systems. These systems not only support cellular growth and alignment but also offer advantages in terms of mechanical integrity, printability, and consumer acceptability, which are key attributes for downstream processing and eventual product formulation.

Although technical challenges remain, including the optimization of scaffold degradation kinetics; the control of mechanical–textural trade-offs; and the standardization of structure–function relationships, integrated approaches that align material selection, fabrication processes, and regulatory frameworks will be essential for advancing edible scaffold technologies toward industrial application. Ultimately, hybrid gel-based scaffolds have the potential to play a central role in enhancing the technological maturity and market readiness of cultured meat, contributing meaningfully to the realization of sustainable, high-value protein production platforms for the future.

## Figures and Tables

**Table 1:** Key requirements for the design of cultured meat scaffolds.

Category	Description	Ref.
Biocompatibility	Requires a biofriendly surface that supports cell adhesion, growth, and differentiation	[7,8,9,10]
Edibility and Safety	Made from food-grade materials that are GRAS-certified, non-toxic, and digestible	[4,11,12]
Mechanical Properties	Elasticity similar to muscle tissue (10–100 kPa); durability to withstand cutting and cooking	[6,13,14]
Porosity and Structure	Pore size (50–200 μm) to enable nutrient/oxygen transport; structure that guides cell alignment	[4,15]
Scalability	Must allow for low-cost, large-scale production without the use of toxic chemicals	[3,5,16]

## Data Availability

No new data were created or analyzed in this study.

## References

[1] Post M.J. (2012). Cultured meat from stem cells: Challenges and prospects. Meat Sci..

[2] Tuomisto H.L. (2018). Importance of considering environmental sustainability in dietary guidelines. Lancet Planet. Health.

[3] Ben-Arye T., Shandalov Y., Ben-Shaul S., Landau S., Zagury Y., Ianovici I., Lavon N., Levenberg S. (2020). Textured soy protein scaffolds enable the generation of three-dimensional bovine skeletal muscle tissue for cell-based meat. Nat. Food.

[4] Bomkamp C., Skaalure S.C., Fernando G.F., Ben-Arye T., Swartz E.W., Specht E.A. (2022). Scaffolding Biomaterials for 3D Cultivated Meat: Prospects and Challenges. Adv. Sci..

[5] Lee M., Choi W., Lee J.M., Lee S.T., Koh W.-G., Hong J. (2024). Flavor-switchable scaffold for cultured meat with enhanced aromatic properties. Nat. Commun..

[6] Lee M., Park S., Choi B., Choi W., Lee H., Lee J.M., Lee S.T., Yoo K.H., Han D., Bang G. (2024). Cultured meat with enriched organoleptic properties by regulating cell differentiation. Nat. Commun..

[7] Liu Y., Gao A., Wang T., Zhang Y., Zhu G., Ling S., Wu Z., Jin Y., Chen H., Lai Y. (2025). Growing meat on autoclaved vegetables with biomimetic stiffness and micro-patterns. Nat. Commun..

[8] Chen Y., Zhang W., Ding X., Ding S., Tang C., Zeng X., Wang J., Zhou G. (2024). Programmable scaffolds with aligned porous structures for cell cultured meat. Food Chem..

[9] Hu X., Park S.-H., Gil E.S., Xia X.-X., Weiss A.S., Kaplan D.L. (2011). The influence of elasticity and surface roughness on myogenic and osteogenic-differentiation of cells on silk-elastin biomaterials. Biomaterials.

[10] Boyan B.D., Lotz E.M., Schwartz Z. (2017). Roughness and Hydrophilicity as Osteogenic Biomimetic Surface Properties. Tissue Eng. Part. A.

[11] Li C.H., Yang I.H., Ke C.J., Chi C.Y., Matahum J., Kuan C.Y., Celikkin N., Swieszkowski W., Lin F.H. (2022). The Production of Fat-Containing Cultured Meat by Stacking Aligned Muscle Layers and Adipose Layers Formed from Gelatin-Soymilk Scaffold. Front. Bioeng. Biotechnol..

[12] Andreassen R.C., Rønning S.B., Solberg N.T., Grønlien K.G., Kristoffersen K.A., Høst V., Kolset S.O., Pedersen M.E. (2022). Production of food-grade microcarriers based on by-products from the food industry to facilitate the expansion of bovine skeletal muscle satellite cells for cultured meat production. Biomaterials.

[13] Su L., Jing L., Zeng X., Chen T., Liu H., Kong Y., Wang X., Yang X., Fu C., Sun J. (2023). 3D-Printed Prolamin Scaffolds for Cell-Based Meat Culture. Adv. Mater..

[14] Wu X.-m., Han W.-m., Hou L.-y., Lin D.-d., Li J.-y., Lin S.-t., Yang J.-p., Liao L., Zeng X.-a. (2024). Glutenin-chitosan 3D porous scaffolds with tunable stiffness and systematized microstructure for cultured meat model. Int. J. Biol. Macromol..

[15] Pfaff B.N., Flanagan C.C., Griffin D.R. (2024). Microporous Annealed Particle (MAP) Scaffold Pore Size Influences Mesenchymal Stem Cell Metabolism and Proliferation Without Changing CD73, CD90, and CD105 Expression over Two Weeks. Adv. Biol..

[16] Xie Y., Cai L., Shijie D., Wang C., Wang J., Ibeogu I.H., Li C., Zhou G.H. (2025). An Overview of Recent Progress in Cultured Meat: Focusing on Technology, Quality Properties, Safety, Industrialization, and Public Acceptance. J. Nutr..

[17] Xia P., Miyajima H., Fujita S. (2025). Development of Biomimetic Edible Scaffolds for Cultured Meat Based on the Traditional Freeze-Drying Method for Ito-Kanten (Japanese Freeze-Dried Agar). Gels.

[18] Dagès B.A.S., Fabian J.A., Polakova D., Rysova M., Topham P.D., Souppez J.-B.R.G., Hanga M.P., Theodosiou E. (2025). Edible electrospun materials for scalable cultivated beef production. Food Bioprod. Process..

[19] Wang Y., Zhong Z., Munawar N., Zan L., Zhu J. (2024). 3D edible scaffolds with yeast protein: A novel alternative protein scaffold for the production of high-quality cell-cultured meat. Int. J. Biol. Macromol..

[20] Nurul Alam A.M.M., Kim C.-J., Kim S.-H., Kumari S., Lee E.-Y., Hwang Y.-H., Joo S.-T. (2024). Scaffolding fundamentals and recent advances in sustainable scaffolding techniques for cultured meat development. Food Res. Int..

[21] Jeong D., Jang G., Jung W.K., Park Y.H., Bae H. (2024). Stretchable zein-coated alginate fiber for aligning muscle cells to artificially produce cultivated meat. npj Sci. Food.

[22] Rather J.A., Akhter N., Ashraf Q.S., Mir S.A., Makroo H.A., Majid D., Barba F.J., Khaneghah A.M., Dar B.N. (2022). A comprehensive review on gelatin: Understanding impact of the sources, extraction methods, and modifications on potential packaging applications. Food Packag. Shelf Life.

[23] Hong S.J., Kim D.H., Ryoo J.H., Park S.M., Kwon H.C., Keum D.H., Shin D.M., Han S.G. (2024). Influence of Gelatin on Adhesion, Proliferation, and Adipogenic Differentiation of Adipose Tissue-Derived Stem Cells Cultured on Soy Protein-Agarose Scaffolds. Foods.

[24] Hersel U., Dahmen C., Kessler H. (2003). RGD modified polymers: Biomaterials for stimulated cell adhesion and beyond. Biomaterials.

[25] Eom K.-H., Jeong D., Choi J.-Y., Gim G.-M., Yum S.-Y., Jin S., Bae H., Jang G. (2025). MSTN knockout enhances the production of MYOD1-mediated steak-type cultivated meat. J. Anim. Sci. Biotechnol..

[26] Wang X., Wang M., Xu Y., Yin J., Hu J. (2024). A 3D-printable gelatin/alginate/ε-poly-l-lysine hydrogel scaffold to enable porcine muscle stem cells expansion and differentiation for cultured meat development. Int. J. Biol. Macromol..

[27] Chae S., Lee J., Kim G. (2019). Skeletal myotube formation enhanced through fibrillated collagen nanofibers coated on a 3D-printed polycaprolactone surface. Colloids Surf. B Biointerfaces.

[28] Liu X., Gao Y., Long X., Hayashi T., Mizuno K., Hattori S., Fujisaki H., Ogura T., Wang D.O., Ikejima T. (2020). Type I collagen promotes the migration and myogenic differentiation of C2C12 myoblasts via the release of interleukin-6 mediated by FAK/NF-κB p65 activation. Food Funct..

[29] Basurto I.M., Mora M.T., Gardner G.M., Christ G.J., Caliari S.R. (2021). Aligned and electrically conductive 3D collagen scaffolds for skeletal muscle tissue engineering. Biomater. Sci..

[30] Yun Y.R., Lee S., Jeon E., Kang W., Kim K.H., Kim H.W., Jang J.H. (2012). Fibroblast growth factor 2-functionalized collagen matrices for skeletal muscle tissue engineering. Biotechnol. Lett..

[31] Xing Q., Yates K., Vogt C., Qian Z., Frost M.C., Zhao F. (2014). Increasing Mechanical Strength of Gelatin Hydrogels by Divalent Metal Ion Removal. Sci. Rep..

[32] Sarrigiannidis S.O., Rey J.M., Dobre O., González-García C., Dalby M.J., Salmeron-Sanchez M. (2021). A tough act to follow: Collagen hydrogel modifications to improve mechanical and growth factor loading capabilities. Mater. Today Bio.

[33] Pires Figueiredo M., Rodríguez-Fernández S., Copes F., Mantovani D. (2025). Review of collagen type I-based hydrogels: Focus on composition-structure-properties relationships. npj Biomed. Innov..

[34] Yang G., Xiao Z., Long H., Ma K., Zhang J., Ren X., Zhang J. (2018). Assessment of the characteristics and biocompatibility of gelatin sponge scaffolds prepared by various crosslinking methods. Sci. Rep..

[35] Iber B.T., Kasan N.A., Torsabo D., Omuwa J.W. (2021). A Review of Various Sources of Chitin and Chitosan in Nature. J. Renew. Mater..

[36] Yadav M., Kaushik B., Rao G.K., Srivastava C.M., Vaya D. (2023). Advances and challenges in the use of chitosan and its derivatives in biomedical fields: A review. Carbohydr. Polym. Technol. Appl..

[37] Taborda M.I., Catalan K.N., Orellana N., Bezjak D., Enrione J., Acevedo C.A., Corrales T.P. (2023). Micropatterned Nanofiber Scaffolds of Salmon Gelatin, Chitosan, and Poly(vinyl alcohol) for Muscle Tissue Engineering. ACS Omega.

[38] Smoak M.M., Hogan K.J., Grande-Allen K.J., Mikos A.G. (2021). Bioinspired electrospun dECM scaffolds guide cell growth and control the formation of myotubes. Sci. Adv..

[39] Kang Y.M., Lee B.N., Ko J.H., Kim G.H., Kang K.N., Kim D.Y., Kim J.H., Park Y.H., Chun H.J., Kim C.H. (2010). In vivo biocompatibility study of electrospun chitosan microfiber for tissue engineering. Int. J. Mol. Sci..

[40] Ma X., Ge J., Li Y., Guo B., Ma P.X. (2014). Nanofibrous electroactive scaffolds from a chitosan-grafted-aniline tetramer by electrospinning for tissue engineering. RSC Adv..

[41] Ardean C., Davidescu C.M., Nemeş N.S., Negrea A., Ciopec M., Duteanu N., Negrea P., Duda-Seiman D., Muntean D. (2021). Antimicrobial Activities of Chitosan Derivatives. Pharmaceutics.

[42] Waibel K.H., Haney B., Moore M., Whisman B., Gomez R. (2011). Safety of chitosan bandages in shellfish allergic patients. Mil. Med..

[43] Tofanica B.-M., Mikhailidi A., Samuil C., Ungureanu O.C., Fortună M.E., Ungureanu E. (2024). Advances in Cellulose-Based Hydrogels: Current Trends and Challenges. Gels.

[44] Liang Q., Zhang D., Ji P., Sheng N., Zhang M., Wu Z., Chen S., Wang H. (2021). High-Strength Superstretchable Helical Bacterial Cellulose Fibers with a “Self-Fiber-Reinforced Structure”. ACS Appl. Mater. Interfaces.

[45] Skogberg A., Mäki A.J., Mettänen M., Lahtinen P., Kallio P. (2017). Cellulose Nanofiber Alignment Using Evaporation-Induced Droplet-Casting, and Cell Alignment on Aligned Nanocellulose Surfaces. Biomacromolecules.

[46] Murugarren N., Roig-Sanchez S., Antón-Sales I., Malandain N., Xu K., Solano E., Reparaz J.S., Laromaine A. (2022). Highly Aligned Bacterial Nanocellulose Films Obtained During Static Biosynthesis in a Reproducible and Straightforward Approach. Adv. Sci..

[47] Santos A.E.A.d., Cotta T., Santos J.P.F., Camargos J.S.F., Carmo A.C.C.d., Alcântara E.G.A., Fleck C., Copola A.G.L., Nogueira J.M., Silva G.A.B. (2023). Bioactive cellulose acetate nanofiber loaded with annatto support skeletal muscle cell attachment and proliferation. Front. Bioeng. Biotechnol..

[48] Mastrodimos M., Jain S., Badv M., Shen J., Montazerian H., Meyer C.E., Annabi N., Weiss P.S. (2024). Human Skeletal Muscle Myoblast Culture in Aligned Bacterial Nanocellulose and Commercial Matrices. ACS Appl. Mater. Interfaces.

[49] Gu Y., Somerville C. (2010). Cellulose synthase interacting protein: A new factor in cellulose synthesis. Plant Signal Behav..

[50] Chen J., Ma A., Zhang Y., Sun L., Yang K., Vanegas Sáenz J.R., Hong G. (2025). Mechanical and biological properties of cellulose nanofibers as a dental biomaterial. J. Dent. Sci..

[51] Kummala R., Soto Véliz D., Fang Z., Xu W., Abitbol T., Xu C., Toivakka M. (2020). Human Dermal Fibroblast Viability and Adhesion on Cellulose Nanomaterial Coatings: Influence of Surface Characteristics. Biomacromolecules.

[52] Cândido A., Fregonezi N., Carvalho A., Trovatti E., Resende F. (2020). TEMPO-Oxidized Cellulose Nanofibers In Vitro Cyto-genotoxicity Studies. BioNanoScience.

[53] Hu Y.J., Wang Y., Huang Y.H., Bian J., Li M.F., Peng F., Sun R.C. (2019). Benzoxazine enhanced amino cellulose-based composite films: Preparation, proposed mechanism, and improved performance. Carbohydr. Polym..

[54] Claro A., Amaral N., Colturato V., Andrade Aleixo N., Paiva R., Cruz S., Monteiro G., Carvalho G., Nogueira F., Deffune E. (2022). Siloxane-modified bacterial cellulose as a promising platform for cell culture. Cellulose.

[55] Ahangari H., Ebrahimi A., Ehsani A., Amjadi S. (2025). Multipurpose packaging system based on intelligent carboxymethyl cellulose film and activated cellulose acetate electrospun nanofibers for seafoods. Int. J. Biol. Macromol..

[56] Vatankhah E., Prabhakaran M.P., Jin G., Mobarakeh L.G., Ramakrishna S. (2014). Development of nanofibrous cellulose acetate/gelatin skin substitutes for variety wound treatment applications. J. Biomater. Appl..

[57] Park S., Jung S., Heo J., Koh W.G., Lee S., Hong J. (2021). Chitosan/Cellulose-Based Porous Nanofilm Delivering C-Phycocyanin: A Novel Platform for the Production of Cost-Effective Cultured Meat. ACS Appl. Mater. Interfaces.

[58] Melzener L., Spaans S., Hauck N., Pötgens A.J.G., Flack J.E., Post M.J., Doğan A. (2023). Short-Stranded Zein Fibers for Muscle Tissue Engineering in Alginate-Based Composite Hydrogels. Gels.

[59] Kawecki N.S., Norris S.C.P., Xu Y., Wu Y., Davis A.R., Fridman E., Chen K.K., Crosbie R.H., Garmyn A.J., Li S. (2023). Engineering multicomponent tissue by spontaneous adhesion of myogenic and adipogenic microtissues cultured with customized scaffolds. Food Res. Int..

[60] Dey S., Hettiarachchy N., Bisly A.A., Luthra K., Atungulu G.G., Ubeyitogullari A., Mozzoni L.A. (2022). Physical and textural properties of functional edible protein films from soybean using an innovative 3D printing technology. J. Food Sci..

[61] Kim W.-J., Lu Y., Ovissipour R., Nitin N. (2024). Evaluation of plant-based composite materials as 3D printed scaffolds for cell growth and proliferation in cultivated meat applications. Food Hydrocoll..

[62] Seo J.W., Jung W.K., Park Y.H., Bae H. (2023). Development of cultivable alginate fibers for an ideal cell-cultivated meat scaffold and production of hybrid cultured meat. Carbohydr. Polym..

[63] Li L., Chen L., Chen X., Chen Y., Ding S., Fan X., Liu Y., Xu X., Zhou G., Zhu B. (2022). Chitosan-sodium alginate-collagen/gelatin three-dimensional edible scaffolds for building a structured model for cell cultured meat. Int. J. Biol. Macromol..

[64] Ahmad Hariza A.M., Mohd Yunus M.H., Fauzi M.B., Murthy J.K., Tabata Y., Hiraoka Y. (2023). The Fabrication of Gelatin-Elastin-Nanocellulose Composite Bioscaffold as a Potential Acellular Skin Substitute. Polymers.

[65] LakshmiBalasubramaniam S., Tajvidi M., Skonberg D. (2024). Hydrophobic corn zein-modified cellulose nanofibril (CNF) films with antioxidant properties. Food Chem..

[66] Zuber J., Lopes Cascabulho P., Gemini Piperni S., Farias Corrêa do Amaral R.J., Vogt C., Carre V., Hertzog J., Kontturi E., Trubetskaya A. (2024). Fast, Easy, and Reproducible Fingerprint Methods for Endotoxin Characterization in Nanocellulose and Alginate-Based Hydrogel Scaffolds. Biomacromolecules.

[67] Wang L., Li T., Wang Z., Hou J., Liu S., Yang Q., Yu L., Guo W., Wang Y., Guo B. (2022). Injectable remote magnetic nanofiber/hydrogel multiscale scaffold for functional anisotropic skeletal muscle regeneration. Biomaterials.

[68] Wan Z., Wang L., Ma L., Sun Y., Yang X. (2017). Controlled Hydrophobic Biosurface of Bacterial Cellulose Nanofibers through Self-Assembly of Natural Zein Protein. ACS Biomater. Sci. Eng..

[69] Bampidis V., Azimonti G., Bastos M., Christensen H., Dusemund B., Durjava M., Kouba M., Lopez-Alonso M., López S., Marcon F. (2020). Safety and efficacy of microcrystalline cellulose for all animal species. EFSA J. Eur. Food Saf. Auth..

[70] Park S.-M., Ryoo J.-H., Kwon H.C., Han S.G. (2025). Scaffold Biomaterials in the Development of Cultured Meat: A Review. Food Sci. Anim. Resour..

[71] Chen Y., Li L., Chen L., Shao W., Chen X., Fan X., Liu Y., Ding S., Xu X., Zhou G. (2023). Gellan gum-gelatin scaffolds with Ca^2+^ crosslinking for constructing a structured cell cultured meat model. Biomaterials.

[72] Vasić K., Knez Ž., Leitgeb M. (2023). Transglutaminase in Foods and Biotechnology. Int. J. Mol. Sci..

[73] Wu X., Liu Y., Liu A., Wang W. (2017). Improved thermal-stability and mechanical properties of type I collagen by crosslinking with casein, keratin and soy protein isolate using transglutaminase. Int. J. Biol. Macromol..

[74] Broderick E.P., O’Halloran D.M., Rochev Y.A., Griffin M., Collighan R.J., Pandit A.S. (2004). Enzymatic stabilization of gelatin-based scaffolds. J. Biomed. Mater. Res. Part B.

[75] Chien K.B., Shah R.N. (2012). Novel soy protein scaffolds for tissue regeneration: Material characterization and interaction with human mesenchymal stem cells. Acta Biomater..

[76] Sengor M. (2022). Transglutaminase crosslinked sodium caseinate/starch/tri Calcium Phosphate based flexible sponge grafts. Mater. Lett..

[77] Lerner A., Matthias T. (2020). Processed Food Additive Microbial Transglutaminase and Its Cross-Linked Gliadin Complexes Are Potential Public Health Concerns in Celiac Disease. Int. J. Mol. Sci..

[78] Choi H., Lee K. (2022). Crosslinking Mechanisms of Phenol, Catechol, and Gallol for Synthetic Polyphenols: A Comparative Review. Appl. Sci..

[79] Taheri P., Jahanmardi R., Koosha M., Abdi S. (2020). Physical, mechanical and wound healing properties of chitosan/gelatin blend films containing tannic acid and/or bacterial nanocellulose. Int. J. Biol. Macromol..

[80] Shokrani H., Shokrani A., Seidi F., Mashayekhi M., Kar S., Nedeljkovic D., Kuang T., Saeb M.R., Mozafari M. (2023). Polysaccharide-based biomaterials in a journey from 3D to 4D printing. Bioeng. Transl. Med..

[81] Amr M., Dykes I., Counts M., Kernan J., Mallah A., Mendenhall J., Van Wie B., Abu-Lail N., Gozen B.A. (2021). 3D printed, mechanically tunable, composite sodium alginate, gelatin and Gum Arabic (SA-GEL-GA) scaffolds. Bioprinting.

[82] Wang Y., Zhang L., Cao G., Li Z., Du M. (2024). Effect of Heat Treatment on Gelatin Properties and the Construction of High Internal Phase Emulsions for 3D Printing. Foods.

[83] Ciobanu M.-M., Manoliu D.-R., Ciobotaru M.C., Flocea E.-I., Boișteanu P.-C. (2025). Dietary Fibres in Processed Meat: A Review on Nutritional Enhancement, Technological Effects, Sensory Implications and Consumer Perception. Foods.

[84] Fraeye I., Kratka M., Vandenburgh H., Thorrez L. (2020). Sensorial and Nutritional Aspects of Cultured Meat in Comparison to Traditional Meat: Much to Be Inferred. Front. Nutr..

[85] Lee J.-Y., Kamel J., Yadav C.-J., Yadav U., Afrin S., Son Y.-M., Won S.-Y., Han S.-S., Park K.-M. (2024). Production of Plant-Based, Film-Type Scaffolds Using Alginate and Corn Starch for the Culture of Bovine Myoblasts. Foods.

[86] Liu P., Dang X., Woo M.W., Chattha S.A., An J., Shan Z. (2022). Feasibility Study of Starch-Based Biomass Incorporated 3D Printed Beef. Starch-Stärke.

[87] Piantino M., Muller Q., Nakadozono C., Yamada A., Matsusaki M. (2025). Towards more realistic cultivated meat by rethinking bioengineering approaches. Trends Biotechnol..

[88] Hubbe M., Sjostrand B., Lestelius M., Håkansson H., Swerin A., Henriksson G. (2024). Swelling of cellulosic fibers in aqueous systems: A Review of chemical and mechanistic factors. BioResources.

[89] Bai J., Ren Y., Fan M., Qian H., Wang L., Wu G., Zhang H., Qi X., Xu M., Rao Z. (2019). Physiological functionalities and mechanisms of β-glucans. Trends Food Sci. Technol..

[90] Xian D., Wu L., Lin K., Liu P., Wu S., Yuan Y., Xie F. (2024). Augmenting corn starch gel printability for architectural 3D modeling for customized food. Food Hydrocoll..

[91] Colussi R., Halal S., Zanella Pinto V., Bartz J., Gutkoski L., Zavareze E., Dias A. (2015). Acetylation of rice starch in an aqueous medium for use in food. LWT.

[92] Xie X., Li X., Lei J., Zhao X., Lyu Y., Mu C., Li D., Ge L., Xu Y. (2020). Oxidized starch cross-linked porous collagen-based hydrogel for spontaneous agglomeration growth of adipose-derived stem cells. Mater. Sci. Eng. C.

[93] Habib M.A., Khoda B. (2022). Rheological Analysis of Bio-ink for 3D Bio-printing Processes. J. Manuf. Process.

[94] Hu X., Zhu C., Hu Z., Shen W., Ji Z., Li F., Guo C. (2024). Effect of zein-pectin composite particles on the stability and rheological properties of gelatin/hydroxypropyl methylcellulose water-water systems. Int. J. Biol. Macromol..

[95] Mo Q., Huang L., Sheng Y., Wei Z., Zhang S., Li Y., Wang X., Wang Y., Lu X., Huang C. (2024). Crosslinking strategy and promotion role of cellulose as a composite hydrogel component for three-dimensional printing—A review. Food Hydrocoll..

